# Referral Criteria for Specialist Palliative Care for Patients With Dementia

**DOI:** 10.1001/jamanetworkopen.2025.10298

**Published:** 2025-05-14

**Authors:** Yuchieh Kathryn Chang, Jennifer Philip, Jenny T. van der Steen, Lieve Van den Block, Allyn Yin Mei Hum, Pedro E. Pérez-Cruz, Carlos Paiva, Masanori Mori, Ping-Jen Chen, Meera R. Agar, Laura Hanson, Catherine J. Evans, David Hui

**Affiliations:** 1 Department of Palliative Care, Rehabilitation and Integrative Medicine, University of Texas MD Anderson Cancer Center, Houston; 2 Department of Medicine, University of Melbourne, Fitzroy, Australia; 3 Department of Palliative Care, St Vincent’s Hospital Melbourne, Fitzroy, Australia; 4 Department of Palliative Care, Peter MacCallum Cancer Centre, Melbourne, Australia; 5 Department of Public Health and Primary Care, Leiden University Medical Center, Leiden, the Netherlands; 6 Department of Primary and Community Care and Radboudumc Alzheimer Center, Nijmegen, the Netherlands; 7 VUB-UGent End-of-Life Care Research Group, Department of Family Medicine and Chronic Care, Vrije Universiteit Brussel, Brussels, Belgium; 8 Department of Palliative Medicine, Tan Tock Seng Hospital, Singapore; 9 The Palliative Care Centre for Excellence in Research and Education, Dover Park Hospice, Singapore; 10 Sección de Medicina Paliativa, Escuela de Medicina, Pontificia Universidad Católica de Chile, Santiago, Chile; 11 Centro para el Control y la Prevención del Cáncer, Santiago, Chile; 12 Department of Clinical Oncology, Barretos Cancer Hospital, Barretos, São Paulo, Brazil; 13 Division of Palliative and Supportive Care, Seirei Mikatahara General Hospital, Hamamatsu, Shizuoka, Japan; 14 Department of Family Medicine and Division of Geriatrics and Gerontology, Kaohsiung Medical University Hospital, Kaohsiung, Taiwan; 15 National Center for Geriatrics and Welfare Research, National Health Research Institutes, Miaoli, Taiwan; 16 School of Medicine, College of Medicine, National Sun Yat-sen University, Kaohsiung, Taiwan; 17 IMPACCT, Faculty of Health, University of Technology Sydney, Sydney, Australia; 18 Division of Geriatric Medicine and Palliative Care Program, University of North Carolina at Chapel Hill; 19 Cicely Saunders Institute of Palliative Care, Policy and Rehabilitation, King’s College London, London, United Kingdom

## Abstract

**Importance:**

Patients with dementia have considerable supportive care needs. Specialist palliative care may be beneficial, but it is unclear which patients are most appropriate for referral and when they should be referred.

**Objective:**

To identify a set of consensus referral criteria for specialist palliative care for patients with dementia.

**Design, Setting, and Participants:**

In this survey study using 3 rounds of Delphi surveys, an international, multidisciplinary panel of clinicians from 5 continents with expertise in the integration of dementia and palliative care were asked to rate 83 putative referral criteria (generated from a previous systematic review and steering committee discussion). Specialist palliative care was defined as an interdisciplinary team consisting of practitioners with advanced knowledge and skills in palliative medicine offering consultative services for specialist-level palliative care in (nonhospice) inpatient, outpatient, community, and home-based settings.

**Main Outcomes and Measures:**

Consensus was defined a priori as at least 70% agreement among experts. A criterion was coded as major if the experts advocated that meeting 1 criterion alone was satisfactory to justify a referral. Data were summarized using descriptive statistics.

**Results:**

Of the 63 invited and eligible panelists, the response rate was 58 (92.1%) in round 1, 58 (92.1%) in round 2, and 60 (95.2%) in round 3. Of the 58 panelists who provided demographic data in round 1, most were aged 40 to 49 years (28 of 58 [48.3%]), and 29 panelists (50%) each were men and women. Panelists achieved consensus on 15 major and 42 minor criteria for specialist palliative care referral. The 15 major criteria were grouped under 5 categories, including dementia type (eg, rapidly progressive dementia), symptom distress (eg, severe physical symptoms), psychosocial factors or decision-making (eg, request for hastened death, assisted suicide, or euthanasia), comorbidities or complications (eg, ≥2 episodes of aspiration pneumonia in the past 12 months); and hospital use (eg, ≥2 hospitalizations within the past 3 months).

**Conclusions and Relevance:**

In this Delphi survey study, international experts reached consensus on a range of criteria for referral to specialist palliative care. With testing and validation, these criteria may be used to standardize specialist palliative care access for patients with dementia across various care settings.

## Key Points

**Question:**

For patients with dementia, who is most appropriate for referral to specialist palliative care, and when should they be referred?

**Findings:**

In this survey study using a Delphi survey administered over 3 iterative rounds, 63 international expert panelists reached consensus on 15 major referral criteria for specialist palliative care.

**Meaning:**

These findings suggest that with further testing and validation, these criteria may be used to standardize specialist palliative care access for patients with dementia across various care settings.

## Teaser

This survey study using a 3-round Delphi survey of international experts identifies a set of consensus referral criteria for specialist palliative care for patients with dementia.

## Introduction

Dementia is an ever-growing public health issue with currently more than 55 million people worldwide living with this disease.^1^ Due to an aging global population, this number is projected to triple by 2050.^2^ Patients with dementia may experience a multitude of symptoms and be at risk for frequent hospitalizations due to disease-related complications and/or complex symptoms throughout the disease trajectory and as they approach the end of life.^3,4,5,6,7^ Specialist palliative care is an interdisciplinary team of trained clinicians in specialist-level palliative medicine who care for patients with life-limiting illnesses and their families by addressing their various physical, psychosocial, and spiritual needs with the aim to improve their quality of life.^8,9^

The World Health Organization stated that specialist palliative care is a component of palliative care service delivery for patients with dementia.^10^ However, specialist palliative care remains limited for patients with dementia and often occurs late in the dementia illness trajectory.^11,12,13^ These limitations may be partly due to the undefined roles of primary palliative care and specialist palliative care, along with the absence of appropriate criteria for referral, as there continues to be an evolution of global palliative care development given the global variability of available resource of, and thus access to, specialist palliative care. A 2021 systematic review found marked heterogeneity in referral criteria for patients with dementia to specialist palliative care.^14^ Though it identified many reasons to involve specialist palliative care, the lack of consensus highlighted the need for further study on a set of consensus referral criteria that may streamline referrals to specialist palliative care in a timely manner to optimize patient and caregiver outcomes.

As specialist palliative care resources are scarce (eg, trained practitioners, specialist palliative care services), a set of simple, robust, and valid criteria may help identify patients who would most likely benefit from specialist palliative care referral (as opposed to primary palliative care alone), thereby improving timely access and resource use.^15,16^ Furthermore, standardized referral criteria may allow programs to develop quality improvement programs, facilitate benchmarking for services delivering dementia care and better define resources to support the growth and education of specialist palliative care programs in this setting, and establish eligibility criteria for future clinical trials involving dementia and palliative care.^17,18,19^ In this study, we aimed to define a set of consensus referral criteria among international experts on specialist palliative care for patients with dementia.

## Methods

This survey study consisted of 3 Delphi survey rounds to identify consensus referral criteria for specialist palliative care for patients with dementia, building on the findings of a recent systematic review.^14^ To ensure diversity throughout the study, we assembled a steering committee of 13 international members from Australia, Belgium, Brazil, Chile, Japan, Singapore, Taiwan, the Netherlands, the UK, and the US and with multidisciplinary backgrounds, including palliative care, geriatrics, and epidemiology. The study was approved by the MD Anderson Cancer Center Institutional Review Board, which waived the need for informed consent as it was not considered human participants research. This study adhered to the American Association for Public Opinion Research (AAPOR) reporting guideline and Conducting and Reporting Delphi Studies guidance, where applicable.^20,21^

### Panelist Selection

Expert panelists consisted of international clinicians with extensive knowledge of dementia and/or palliative care through their training, clinical practice, and/or research. Panelists needed to fulfill all 4 of the following eligibility criteria: (1) be a clinician (physician, advanced practice practitioner, nurse practitioner, or consultant) with an active (at least 20% clinical) specialty clinical practice in geriatrics, neurology, psychiatry, and/or palliative care and at least 5 years of postqualification clinical experience working with patients with dementia; (2) work at a center with access to specialist palliative care services ([nonhospice] inpatient, outpatient/ambulatory clinic, and/or community-based specialist palliative care); (3) have at least board certification or equivalent in both palliative care and either geriatrics, neurology, psychiatry, publications in the area of integration of palliative care and dementia in the past 10 years, or involvement in national or international palliative care guideline development on the topic of integration; and (4) be able to communicate in English. These criteria were strictly delineated to ensure that panelists had a high level of expertise relevant to the study question.

Initial identification of potentially eligible panel candidates was established through a previous systematic review^14^ of the literature that examined referral criteria for palliative care among patients with dementia, professional societies in dementia, and recommendations from our Delphi study steering committee members. To ensure representation from different regions, we purposely sampled for experts in 5 continents (Asia, Australasia, Europe, North America, and South America). Potentially eligible candidates were contacted via an invitation email listing the aforementioned eligibility criteria and outlining the study process. Snowball sampling was also used in which these potential candidates were asked to nominate other experts known to them, who we then contacted to assess eligibility. All fully eligible, interested experts were invited to participate in this Delphi study and sent the surveys.

### Process

Our Delphi study consisted of 3 online survey rounds (Cognito Forms; Cognito LLC), each lasting approximately 3 to 4 weeks, spaced 5 to 6 weeks apart between rounds 1 and 2 and 7 to 8 weeks apart between rounds 2 and 3 due to the holidays (Figure). Nonrespondents were sent weekly reminder emails after weeks 1 and 2, followed by a final email reminder in week 3. No financial incentives were provided. Prior to each Delphi round, the steering committee members reviewed and revised the referral criteria list, Delphi survey format, and specific wording used. For this study, specialist palliative care was defined as an interdisciplinary team consisting of practitioners with advanced knowledge and skills in palliative medicine offering consultative services for specialist-level palliative care in an inpatient, outpatient, community, and/or home-based setting (nonhospice).^8^

**Figure.**
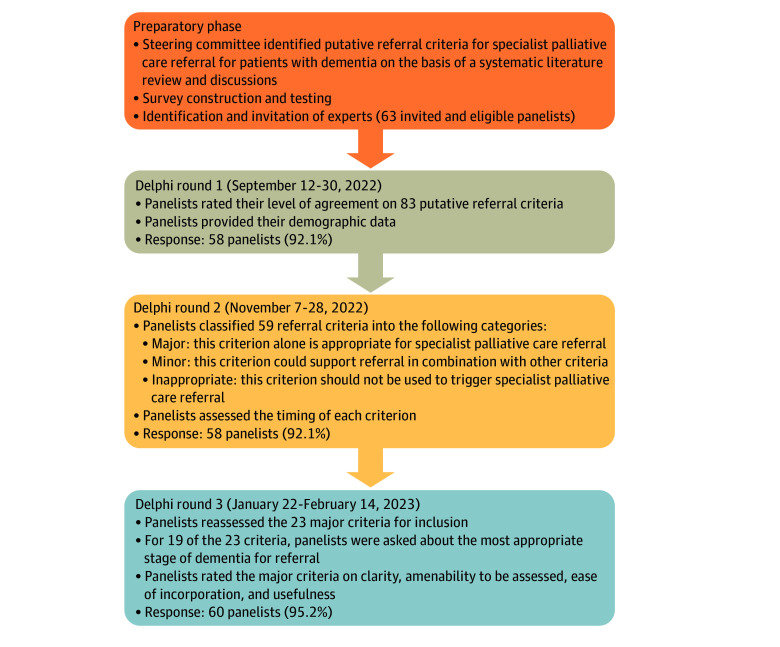
Delphi Process for Reaching International Consensus for Specialist Palliative Care Referral in Patients With Dementia

Delphi round 1, conducted September 12 to 30, 2022, consisted of a list of 83 putative referral criteria. We generated this initial list based on criteria previously identified in a systematic review^14^ and discussion among the steering committee members. These referral criteria were grouped under 7 initial categories, including dementia stage and atypical dementia, time-based factors, symptom distress (eg, pain, anxiety, delirium), functional impairment (eg, Functional Assessment Staging Tool, Palliative Performance Scale, Global Deterioration Scale, Clinical Frailty Scale), psychosocial factors or decision-making (eg, financial distress, family/caregiver distress or burden), comorbidities or complications, and hospital use for dementia and/or related complications or symptoms.

Survey respondents (hereafter panelists) were asked to indicate their agreement with the statement, “Specialist palliative care referral should be considered for patients with dementia who meet the following criteria: [insert specific criterion],” using a 5-point Likert scale (strongly agree, agree, neutral, disagree, strongly disagree). For the time-based factors exclusively, we also used a 5-point Likert scale, but asked panelists to rate the timing (too early, relatively early, neither early nor late, relatively late, too late). For each criterion listed, consensus agreement was defined a priori as at least 70% based on previous Delphi studies^17,22,23^ (ie, strongly agree and agree together ≥70%). Basic demographic information was also collected, including age group, sex, practice type, specialty, years of experience, and continent.

Delphi round 2, conducted November 7 to 28, 2022, was formatted based on panelists’ feedback from round 1, as well as the steering committee’s input. This round of the survey revolved around 2 major themes: (1) major and minor criteria for referral and (2) timing based on referral criteria. We included in round 2 all criteria from round 1 that reached at least 50% agreement (59 of 83 criteria). With the time-based criteria, the minimum 50% threshold (to be included in round 2) was based on the combination of relatively early and neither early nor late from the round 1 survey as they were believed to be the 2 most appropriate responses. Each criterion listed also included the percentage agreement from round 1 (among those who participated). The panelists were asked to rate each referral criterion as a major criterion for referral, a minor criterion for referral, or inappropriate for referral. A criterion was coded as major if the panelists agreed that patients who meet this single criterion alone are appropriate for referral. A criterion was coded as minor if it did not qualify as a major criterion but the panelists agreed that patients who meet this criterion plus at least another minor criterion would be appropriate for referral.^17,23^ A criterion was coded as inappropriate if not considered to be a criterion for referral.

Delphi round 3, conducted January 22 to February 14, 2023, was a targeted survey in which we asked the panelists to confirm the validity of each of the 23 major criteria that were identified from round 2 (based on ≥70% agreement for being a major criterion). The panelists were asked to rate their level of agreement from strongly disagree to strongly agree. Each criterion listed included the percentage agreement from round 2. In addition, at the end of each section, we included a box for additional comments. Several panelists from round 2 commented on the necessity of context (eg, stage of dementia) in which to interpret the use of these referral criteria. We therefore elicited responses regarding panelists’ opinions about the most appropriate stages (severe/advanced stage of dementia only; moderate/middle or severe/advanced stage of dementia; any stage of dementia [mild/early, moderate/middle, severe/advanced]) at which specialist palliative care referral would be indicated for 19 of the 23 major criteria.

We also included 2 additional sections. First, we asked panelists to rate on a numeric scale of 0 (not at all) to 10 (very much) whether the major criteria surveyed were clearly stated, can be assessed accurately, can be easily incorporated into routine screening in clinical practice, and can be useful to facilitate specialist palliative care referral in their respective clinical settings. Second, we elicited panelists’ attitudes and beliefs about specialist palliative care referral for patients with dementia by asking them to rate 7 different statements on a 5-point Likert scale (strongly agree, agree, neutral, disagree, strongly disagree). These 7 statements examined their attitudes and beliefs about lateness of referral, referral despite prognosis of more than 24 months, referral based solely on dementia stage regardless of meeting other referral criteria, and whether specialist palliative care teams should receive either basic or formal training on dementia care.

### Statistical Analysis

We summarized the data using descriptive statistics, including counts, frequencies, and percentages. The analysis was performed using Microsoft Excel, version 2503 (Microsoft Corp).

## Results

### Panelist Characteristics

Among the 63 expert panelists identified, 58 (92.1%) responded in round 1, 58 (92.1%) in round 2, and 60 (95.2%) in round 3. Of the 58 panelists who provided demographic data in round 1, most were aged 40 to 49 years (28 [48.3%] vs 6 [10.3%] aged 30-39 years, 18 [31.0%] aged 50-59 years, and 6 [10.3%] aged ≥60 years), 29 each (50%) were female and male, and there was representation from Asia (16 panelists [27.6%]), Europe/UK (14 panelists [24.1%]), South America (12 panelists [20.7%]), North America (9 panelists [15.5%]), and Australasia (7 panelists [12.1%]). Forty-four panelists (75.9%) had received palliative care training, while 35 (60.3%) had received geriatrics training (Table 1). The panelists reported a median (IQR) of 15 (11-23) years’ experience in geriatrics, neurology, or psychiatry or 12 (8-17) years’ experience in palliative care.

**Table 1.** Demographics of Delphi Study Expert Panelists (n = 58)

Characteristic	Panelists, No. (%)
Age group, y	
30-39	6 (10.3)
40-49	28 (48.3)
50-59	18 (31.0)
60-69	4 (6.9)
≥70	2 (3.4)
Sex	
Female	29 (50.0)
Male	29 (50.0)
Continent	
Asia	16 (27.6)
Australasia	7 (12.1)
Europe/UK	14 (24.1)
North America	9 (15.5)
South America	12 (20.7)
Practice setting	
Tertiary care hospital	44 (75.9)
Primary or secondary care hospital	10 (17.2)
Long-term care facility	11 (19.0)
Skilled nursing facility	4 (6.9)
Home care	14 (24.1)
Outpatient/ambulatory clinic	12 (20.7)
Other^a^	7 (12.1)
Specialist palliative care access	
Inpatient	55 (94.8)
Outpatient/ambulatory clinic	33 (56.9)
Community based care	31 (53.4)
Home-based care	36 (62.1)
Other^b^	2 (3.4)
Specialty	
Geriatrics	35 (60.3)
Neurology	6 (10.3)
Psychiatry	5 (8.6)
Palliative care	44 (75.9)
Other^c^	5 (8.6)
Profession	
Physician	55 (94.8)
Advanced practice practitioner	3 (5.2)
Geriatric, neurology, or psychiatry experience, median (IQR), y	15 (11-23)
Palliative care experience, median (IQR), y	12 (8-17)
Expertise	
Board certification or equivalent in both palliative care and either geriatrics, neurology, or psychiatry	39 (66.1)
Published in the area of integration of palliative care and dementia in the past 10 y	29 (49.2)
Have been involved in national or international palliative care guideline development on the topic of integration	26 (44.1)

^a^ For example, palliative care unit or community care.

^b^ For example, senior day care centers or day rehabilitation centers.

^c^ For example, anesthesiology, family medicine, or internal medicine.

### Round 1 and Round 2 Delphi Surveys

In Delphi survey round 1, the panelists reached consensus on 42 of the 83 referral criteria (50.6%) (Table 2). The referral criteria with the highest consensus at 98.3% (57 panelists) was severe physical symptoms, followed by request for hastened death, assisted suicide, or euthanasia at 93.1% (54 panelists) and 91.4% (53 panelists) each for rapidly progressive dementia, hospice referral or discussion, patient or family request, and withdrawal or de-escalation of life-prolonging interventions. In round 2, panelists reached consensus on 57 of 59 referral criteria (96.6%), including 23 classified as major criteria (40.4%) and 34 classified as minor criteria (59.6%). Of the 23 major criteria, only 2 had consensus agreement of 90% or higher, including rapidly progressive dementia (91.4% [53 panelists]) and severe physical symptoms (93.1% [54 panelists]). Two criteria did not meet the set threshold for specialist palliative care referral (dementia from head trauma and physician-estimated life expectancy of >24 months).

**Table 2.** Level of Agreement on Putative Criteria for Specialist Palliative Care Referral for All 3 Delphi Survey Rounds

Criterion	Panelists, No. (%)			
	Round 1 agreement (n = 58)^a^	Round 2 agreement (n = 58)^b^		Round 3 agreement (n = 60)^c^
		Major criterion	Minor criterion	
**Dementia stage and atypical dementia**				
At initial diagnosis of dementia	10 (17.2)	NA	NA	NA
At mild/early stage of dementia	11 (19.0)	NA	NA	NA
At moderate/middle stage of dementia	32 (55.2)	7 (12.1)	41 (70.7)	NA
At severe/advanced stage of dementia^d^	50 (86.2)	46 (79.3)	12 (20.7)	43 (71.7)
Early-onset dementia (age of onset <65 y)	34 (58.6)	11 (19.0)	37 (63.8)	NA
Rapidly progressive dementia	53 (91.4)	53 (91.4)	5 (8.6)	55 (91.7)
Dementia from head trauma	29 (50.0)	4 (6.9)	36 (62.1)	NA
**Time-based factors^e^**				
Physician-estimated life expectancy, mo				
>24	38 (65.5)	1 (1.7)	31 (53.4)	NA
≤24	49 (84.5)	19 (32.8)	35 (60.3)	NA
≤12	40 (69.0)	43 (74.1)	15 (25.9)	37 (61.7)
≤6	25 (43.1)	NA	NA	NA
≤3	12 (20.7)	NA	NA	NA
≤1	6 (10.3)	NA	NA	NA
**Symptom distress**				
Physical symptoms				
Mild	9 (15.5)	NA	NA	NA
Moderate	41 (70.7)	13 (22.4)	43 (74.1)	NA
Severe	57 (98.3)	54 (93.1)	4 (6.9)	60 (100)
Emotional symptoms				
Mild	11 (19.0)	NA	NA	NA
Moderate	33 (56.9)	9 (15.5)	42 (72.4)	NA
Severe	49 (84.5)	48 (82.8)	9 (15.5)	53 (88.3)
Behavioral or neuropsychiatric symptoms				
Mild	12 (20.7)	NA	NA	NA
Moderate	35 (60.3)	13 (22.4)	38 (65.5)	NA
Severe	48 (82.8)	46 (79.3)	11 (19.0)	50 (83.3)
Spiritual or existential distress				
Mild	18 (31.0)	NA	NA	NA
Moderate	41 (70.7)	18 (31.0)	36 (62.1)	NA
Severe	50 (86.2)	50 (86.2)	8 (13.8)	56 (93.3)
**Functional impairment**				
Dependent in ≥3 basic ADL	29 (50.0)	12 (20.7)	34 (58.6)	NA
Upon placement into nursing home or long-term care facility	41 (70.7)	23 (39.7)	31 (53.4)	NA
Functional Assessment Staging Tool stage^f^				
4	10 (17.2)	NA	NA	NA
5	15 (25.9)	NA	NA	NA
6	35 (60.3)	10 (17.2)	40 (69.0)	NA
7	46 (79.3)	41 (70.7)	14 (24.1)	37 (61.7)
Palliative Performance Scale score^g^				
80	5 (8.6)	NA	NA	NA
70	9 (15.5)	NA	NA	NA
60	14 (24.1)	NA	NA	NA
50	34 (58.6)	10 (17.2)	38 (65.5)	NA
40	44 (75.9)	24 (41.4)	27 (46.6)	NA
30	47 (81.0)	39 (67.2)	18 (31.0)	NA
20	45 (77.6)	41 (70.7)	16 (27.6)	39 (65.0)
10	46 (79.3)	41 (70.7)	16 (27.6)	41 (68.3)
Global Deterioration Scale stage^h^				
4	9 (15.5)	NA	NA	NA
5	26 (44.8)	NA	NA	NA
6	46 (79.3)	28 (48.3)	28 (48.3)	NA
7	48 (82.8)	42 (72.4)	15 (25.9)	37 (61.7)
Clinical Frailty Scale score^i^				
5	9 (15.5)	NA	NA	NA
6	23 (39.7)	NA	NA	NA
7	45 (77.6)	26 (44.8)	29 (50)	NA
8	49 (84.5)	40 (69)	17 (29.3)	NA
9	47 (81.0)	44 (75.9)	13 (22.4)	38 (63.3)
**Psychosocial factors or decision-making**				
Severe financial distress	17 (29.3)	NA	NA	NA
Family/caregiver distress/burden	43 (74.1)	31 (53.4)	27 (46.6)	NA
Health care professional distress	44 (75.9)	32 (55.2)	24 (41.4)	NA
Inadequate social support	31 (53.4)	14 (24.1)	28 (48.3)	NA
History of drug or alcohol abuse	24 (41.4)	NA	NA	NA
Assistance with advance care planning	51 (87.9)	35 (60.3)	22 (37.9)	NA
Establish goals of care	50 (86.2)	40 (69.0)	17 (29.3)	NA
Patient and/or family decline to seek care at acute care facilities	50 (86.2)	41 (70.7)	16 (27.6)	45 (75)
Hospice referral or discussion	53 (91.4)	48 (82.8)	9 (15.5)	56 (93.3)
Request for hastened death, assisted suicide, or euthanasia	54 (93.1)	49 (84.5)	7 (12.1)	57 (95.0)
Patient or family request	53 (91.4)	46 (79.3)	12 (20.7)	56 (93.3)
**Comorbidities or complications**				
Multimorbidity	48 (82.8)	35 (60.3)	22 (37.9)	NA
1 Episode of aspiration pneumonia in the past 12 mo	31 (53.4)	13 (22.4)	35 (60.3)	NA
≥2 Episodes of aspiration pneumonia in the past 12 mo	51 (87.9)	41 (70.7)	17 (29.3)	44 (73.3)
Recurrent infections	47 (81.0)	34 (58.6)	23 (39.7)	NA
Chronic skin breakdown (decubitus ulcers stages 3 and 4)	42 (72.4)	28 (48.3)	27 (46.6)	NA
Recurrent falls	31 (53.4)	4 (6.9)	42 (72.4)	NA
Hip fracture	31 (53.4)	12 (20.7)	36 (62.1)	NA
Persistent or worsening dysphagia	49 (84.5)	38 (65.5)	19 (32.8)	NA
Artificial means of nutrition (ie, feeding tube)	48 (82.8)	42 (72.4)	13 (22.4)	44 (73.3)
Chronic hypoalbuminemia	28 (48.3)	NA	NA	NA
Cachexia	38 (65.5)	22 (37.9)	31 (53.4)	NA
Polypharmacy (ie, prescribing behavior, medication review, and reduction)	24 (41.4)	NA	NA	NA
Failure to improve despite optimal medical management	45 (77.6)	36 (62.1)	21 (36.2)	NA
Shock with transfer to ICU	50 (86.2)	41 (70.7)	15 (25.9)	40 (66.7)
Withdrawal or de-escalation of life-prolonging interventions	53 (91.4)	48 (82.8)	8 (13.8)	54 (90.0)
**Hospital use (for dementia and/or related complications or symptoms)**				
≥2 ED visits				
Within the past 3 mo	50 (86.2)	42 (72.4)	14 (24.1)	46 (76.7)
Within the past 6 mo	43 (74.1)	21 (36.2)	31 (53.4)	NA
Within the past 12 mo	23 (39.7)	NA	NA	NA
≥2 Hospitalizations				
Within the past 3 mo	51 (87.9)	41 (70.7)	17 (29.3)	48 (80)
Within the past 6 mo	46 (79.3)	23 (39.7)	30 (51.7)	NA
Within the past 12 mo	25 (43.1)	NA	NA	NA
≥1 ICU admission				
Within the past 3 mo	51 (87.9)	42 (72.4)	14 (24.1)	46 (76.7)
Within the past 6 mo	46 (79.3)	33 (56.9)	20 (34.5)	NA
Within the past 12 mo	32 (55.2)	10 (17.2)	35 (60.3)	NA

Abbreviations: ADL, activities of daily living; ED, emergency department; ICU, intensive care unit; NA, not applicable.

^a^ The number of respondents who strongly agreed or agreed with the stated criterion being considered a potential trigger for specialist palliative care referral.

^b^ Included are only criteria from round 1 that reached at least 50% agreement (59 of 83 criteria; 23 classified as major, and 34 classified as minor).

^c^ Round 3 was conducted to confirm the validity of each of the 23 major criteria identified from round 2 (based on ≥70% agreement for being a major criterion).

^d^ Even though the criterion at severe/advanced stage of dementia reached 71.7% agreement in round 3, it was designated as a minor criterion because the same expert panel only reached 48.3% agreement for the statement, “Patients with advanced stage dementia should be referred to specialist palliative care within 3 months of entering this stage, regardless of whether they meet any other referral criteria,” when further clarification was sought (eTable in Supplement 1).

^e^ With the time-based criteria, the minimum 50% threshold (to be included in round 2) was based on the combination of relatively early and neither early nor late from the round 1 survey, as they were believed to be the 2 most appropriate responses.

^f^ Higher stages indicate a more advanced stage of cognitive decline.

^g^ Higher scores indicate better functional status.

^h^ Higher stages indicate a more severe stage of cognitive decline in patients with dementia.

^i^ Higher scores indicate increase frailty.

### Round 3 Delphi Survey

Of the 23 referral criteria presented in Delphi survey round 3, there were 7 that did not reach a final consensus of at least 70% (Table 3). Even though the criterion of at severe/advanced stage of dementia reached 71.7% agreement in round 3, it was designated as a minor criterion because the same expert panel only reached 48.3% agreement for the statement, “Patients with advanced stage dementia should be referred to specialist palliative care within 3 months of entering this stage, regardless of whether they meet any other referral criteria,” when we sought further clarification (eTable in Supplement 1). The final 15 major referral criteria (Table 4) were grouped under the following 5 categories: symptom distress, psychosocial factors or decision-making, dementia type, comorbidities or complications, and hospital use for dementia and/or related complications or symptoms. Only 1 criterion, severe physical symptoms, reached 100% consensus (60 panelists).

**Table 3.** Delphi Round 3 Major Referral Criteria for Specialist Palliative Care for Patients With Dementia Along With Appropriate Dementia Stage to Consider Referral (n = 60)^a^

Referral criterion	Panelists, No. (%)^b^^,^^c^		
	Severe dementia only	Moderate or severe dementia	Any stage of dementia
Severe physical symptoms	10 (16.7)	27 (45.0)	23 (38.3)
Severe emotional symptoms	19 (31.7)	23 (38.3)	18 (30.0)
Severe behavioral or neuropsychiatric symptoms	23 (38.3)	17 (28.3)	20 (33.3)
Severe spiritual or existential distress	12 (20.0)	21 (35.0)	27 (45.0)
Patient and/or family decline to seek care at acute care facilities	15 (25.0)	25 (41.7)	20 (33.3)
Hospice referral or discussion	11 (18.3)	27 (45.0)	22 (36.7)
Request for hastened death, assisted suicide, or euthanasia	8 (13.6)	15 (25.4)	36 (61.0)
Patient or family request	10 (16.7)	18 (30.0)	32 (53.3)
Rapidly progressive dementia	6 (10.0)	19 (31.7)	35 (58.3)
≥2 Episodes of aspiration pneumonia in the past 12 mo	18 (30.0)	26 (43.3)	16 (26.7)
Artificial means of nutrition (ie, feeding tube)	19 (31.7)	26 (43.3)	15 (25.0)
Withdrawal or de-escalation of life-prolonging interventions	11 (18.3)	24 (40.0)	25 (41.7)
≥2 ED visits within the past 3 mo	23 (38.3)	28 (46.7)	9 (15.0)
≥2 Hospitalizations within the past 3 mo	22 (36.7)	28 (46.7)	10 (16.7)
≥1 ICU admission within the past 3 mo	18 (30.0)	31 (51.7)	11 (18.3)

Abbreviations: ED, emergency department; ICU, intensive care unit.

^a^ To determine the most appropriate stages of dementia to trigger a specialist palliative care referral for each major criterion, whether any stage reached at least 70% was examined. If not, then the combination of moderate or severe stage and any stage reaching at least 70% was examined. If yes, then the major criterion would be appropriate for any patient with moderate or severe dementia. If not, then the criterion would be only appropriate for a patient with severe dementia.

^b^ Seven criteria not reaching consensus for major criteria in round 3 included physician-estimated life expectancy of 12 months or less; Functional Assessment Staging Tool stage 7; Palliative Performance Scale score of 20; Palliative Performance Scale score of 10; Global Deterioration Scale stage 7; Clinical Frailty Scale score of 9; and shock with transfer to the ICU.

^c^ Even though the criterion at severe/advanced stage of dementia reached 71.7% agreement in round 3, it was designated as a minor criterion because the same expert panel only reached 48.3% agreement for the statement, “Patients with advanced stage dementia should be referred to specialist palliative care within 3 months of entering this stage, regardless of whether they meet any other referral criteria,” when further clarification was sought (eTable in Supplement 1).

**Table 4.** Final Major Referral Criteria for Specialist Palliative Care and at Which Stage of Dementia to Consider Referral

Criterion	Stage of dementia
**Needs-based**	
Symptom distress^a^	
Severe physical symptoms	Moderate
Severe spiritual or existential distress	Moderate
Severe emotional symptoms	Severe
Severe behavioral or neuropsychiatric symptoms	Severe
Psychosocial factors or decision making	
Request for hastened death, assisted suicide, euthanasia^b^	Moderate
Hospice referral or discussion^b^	Moderate
Patient or family request	Moderate
Patient and/or family decline to seek care at acute care facilities	Moderate
**Disease-based**	
Dementia type	
Rapidly progressive dementia	Moderate
Comorbidities or complications	
Withdrawal or de-escalation of life-prolonging interventions	Moderate
≥2 Episodes of aspiration pneumonia in the past 12 mo	Moderate
Artificial means of nutrition (ie, feeding tube)	Severe
Hospital use^c^	
≥1 ICU admission within the past 3 mo	Moderate
≥2 ED visits within the past 3 mo	Severe
≥2 Hospitalizations within the past 3 mo	Severe

Abbreviations: ED, emergency department; ICU, intensive care unit.

^a^ Refractory to primary palliative care interventions.

^b^ May not be applicable to all countries.

^c^ For dementia and/or related complications or symptoms.

The panelists rated these major referral criteria as being clearly stated (median [IQR], 8 [7-9] points), can be assessed accurately (median [IQR], 8 [7-9] points), easily incorporated into routine screening in clinical practice (median [IQR], 7 [6-8] points), and useful to facilitate specialist palliative care referral in their respective clinical settings (median [IQR], 8 [6-8] points).

Panelists reached consensus of at least 70% on 4 of the 7 statements about attitudes and beliefs regarding specialist palliative care referral (eTable in Supplement 1). At 70.0% agreement (42 panelists), the panelists believed that if a patient with dementia meets any of the major criteria, they should be referred to specialist palliative care even if their life expectancy is more than 24 months. Seventy-five percent (45 panelists) believed that patients with dementia were being referred to specialist palliative care too late in the disease process in their respective clinical practice settings. When it came to training, the majority (57 panelists [95.0%]) supported the belief that specialist palliative care teams delivering care to patients with dementia should, at a minimum, receive basic training (eg, informal didactic lectures) in dementia, and 81.7% (49 panelists) believed that formal mandatory training (eg, supervised clinical training/rotation) is needed.

## Discussion

In this survey study using 3 rounds of Delphi surveys, the large number of criteria (15 major and 42 minor) that reached consensus suggests that the expert panelists recognized the many opportunities for specialist palliative care involvement to enhance dementia care beyond primary palliative care. The 15 major criteria fell under 5 categories and can be broadly classified as needs-based criteria (ie, symptom distress and psychosocial factors or decision-making) and disease-based criteria (ie, dementia type, comorbidities or complications, and hospital use). With needs-based criteria, palliative care teams have specialized communication training and resources to help patients navigate the complex decisions surrounding end-of-life care planning, such as hospice referral and addressing requests for hastened death, assisted suicide, or euthanasia, especially in countries in which these practices are available and legal. With disease-based criteria, for example, patients with rapidly progressive dementia, such as prion disease, may have a range of needs, from symptom management to meeting psychosocial needs and engaging in difficult decision-making processes, that may have to occur in a short time span and thus prove to be complex. This arena is where specialist palliative care teams, in coordination with dementia specialists, may help provide the necessary support. With further testing and validation, the 15 major criteria may serve as triggers for specialist palliative care referral for patients with dementia in clinical and research settings.

One pressing question is whether the major referral criteria are only applicable for patients with an advanced stage of dementia or earlier stages. The expert panelists stated that 6 of 8 needs-based criteria and 4 of 7 disease-based criteria were appropriate starting at the moderate stage, with the rest applicable only for patients at the severe stage. For example, patients with refractory physical symptoms, even though they are not in a severe stage of dementia, should be referred to specialist palliative care to address their specific needs while continuing overall care with the referring physician. Other criteria, such as artificial means of nutrition (ie, feeding tube), tend to occur when dementia is severe, and thus, referral at this stage is appropriate. Interestingly, there was no consensus on criteria appropriate for early-stage dementia, reflecting the panelists’ predilection for a proper balance between primary and specialist palliative care roles. With the majority of the final 15 major referral criteria being applicable in the moderate stage of dementia, this consensus highlights support for earlier referral than currently practiced.^11,12,13^

Some patients may not meet any of the major criteria, but this does not necessarily mean that they are not appropriate for specialist palliative care involvement. We identified 42 minor referral criteria that may, in combination, be considered as potential triggers for specialist palliative care referral. Future research needs to examine how to best incorporate these minor criteria and whether their use might result in earlier referral.

Of interest, the expert panelists did not reach consensus on functional impairment or time-based factors or family/caregiver distress/burden as major referral criteria. This finding is somewhat surprising as functional impairment and time-based factors are commonly considered in the literature and family/caregiver distress/burden is an area that specialist palliative care is known to focus on.^24,25,26,27,28,29,30,31^ Potential explanations may be that estimating survival can be difficult in patients with dementia, functional scale scores could fluctuate with disease status, and identification of specific cutoffs for referral is challenging.^31^ Furthermore, there may be differences in opinion on the relevance of functional scale scores given the panelists’ various subspecialties and variations in available resources and/or cultural/societal experiences and norms; thus, they may believe that the other criteria were more able to capture the key factors for referral.

As the number of people with dementia continues to grow worldwide, there is a need and an opportunity to collaborate and provide seamless integration of specialist palliative care with the primary palliative care already delivered^32^ to enhance the care of patients with dementia. To help advance this goal, the 15 major criteria identified here represent a first step toward clarification of primary and specialist palliative care roles and consideration for systematic screening of symptom and supportive care needs.^19^ These criteria are not intended to replace but, rather, to support clinical judgment.^33^ Further research is necessary not only to validate their use in the primary and specialist care settings but also to evaluate for possible impediments to effective implementation. Furthermore, taking into consideration the large number of criteria alone, there may be challenges with operationalization. Consideration of the varied clinical practice settings in differing health care cultures along with the local availability of palliative care resources may necessitate modification of these criteria. Nevertheless, having a set of predefined, validated referral criteria, along with appropriate cutoffs, may allow for optimal allocation of scarce specialist palliative care resources and aid with clarifying the roles of primary and specialist palliative care and, thus, the referral of appropriate patients to specialist palliative care in a timely manner.

### Limitations

Our study has several limitations. First, our multidisciplinary group of expert panelists may not be representative of all the practices of specialist palliative care across the specialties of geriatrics, neurology, psychiatry, and specialist palliative care globally. Second, our sample size was small; however, the panelists were selected for having a thorough knowledge of the topic and a good grasp of the realities of clinical practice. Third, a Delphi study, by design, is based on the opinions of experts and, thus, may give rise to bias. However, to help mitigate this risk, we attempted, through careful selection, to ensure a diverse panel of experts and anonymized participant responses. Finally, though our expert panel included specialists from 5 continents, we were not able to recruit representation from Africa. Even with the multinational representation, our findings are based on panelists mostly from high-income countries and, thus, may not be generalizable to countries or settings with limited specialist palliative care resources. Future studies should more fully examine whether these criteria are applicable in those settings, as well as focus on modification and implementation to effectively use these criteria as a screening tool in primary and specialist care practices.

## Conclusions

In this Delphi survey study, many reasons were highlighted to involve specialist palliative care earlier in dementia care, including starting at the moderate stage, along with the many opportunities for close collaboration between dementia care and specialist palliative care teams. Due to an aging global population, the prevalence of dementia is expected to grow, and the criteria from this Delphi study may forward the advancement of specialist palliative care integration in global dementia care by helping to define primary and specialist palliative care roles, improve standardization of clinical care, and provide the necessary baseline for much-needed clinical trials. Future research is needed to further examine the applicability, modification, and implementation of these criteria across varied clinical practice settings in differing health care cultures and, ultimately, the impact this may have.

## Notes

Supplement 1. **eTable.** Attitudes and Beliefs Regarding Specialist Palliative Care Referral for Patients With Dementia

Supplement 2.
Data Sharing Statement
