# Supplementary material for: Referral Criteria for Specialist Palliative Care for Patients With Dementia
Source: JAMA Netw Open. 2025 May 14;8(5):e2510298. doi: 10.1001/jamanetworkopen.2025.10298 (PMC12079294; doi:10.1001/jamanetworkopen.2025.10298)
Supplement: Supplement 1. — eTable. Attitudes and Beliefs Regarding Specialist Palliative Care Referral for Patients With Dementia [file jamanetwopen-e2510298-s001.pdf]

Supplemental Online Content

Chang YK, Philip J, van der Steen JT, et al. Referral criteria for specialist palliative care for patients with dementia. *JAMA Netw Open*. 2025;8(5):e2510298.
doi:10.1001/jamanetworkopen.2025.10298

eTable. Attitudes and Beliefs Regarding Specialist Palliative Care Referral for Patients With Dementia Results of quality assessment per study

This supplemental material has been provided by the authors to give readers additional information about their work.

© 2025 Chang YK et al. *JAMA Network Open*.

eTable. Attitudes and Beliefs Regarding Specialist Palliative Care Referral for Patients With Dementia

	**% Agreement (n=60)**
In my opinion, patients with dementia in my clinical setting are being referred to specialist palliative care too late in the disease process.	75
If a patient with dementia meets any of the major criteria (e.g. severe symptom distress), they should be referred to specialist palliative care even if their life expectancy is over 24 months.	70
Patients with ADVANCED stage dementia should be referred to specialist palliative care within 3 months of entering this stage, regardless of whether they meet any other referral criteria.	48.3
Patients with MODERATE stage of dementia should be referred to specialist palliative care referral within 3 months of entering this stage, regardless of whether they meet any other referral criteria.	25
Patients with MILD stage of dementia should be referred to specialist palliative care within 3 months of entering this stage, regardless of whether they meet any other referral criteria.	11.7
Specialist palliative care teams providing care to patients with dementia should, at a minimum, receive basic training (e.g., informal didactic lectures) in dementia.	95
Specialist palliative care teams providing care to patients with dementia should receive formal mandatory training (e.g., supervised clinical training/rotation) in dementia.	81.7

© 2025 Chang YK et al. *JAMA Network Open*.
