# Supplementary material for: Referral Criteria for Specialist Palliative Care for Patients With Dementia
Source: JAMA Netw Open. 2025 May 14;8(5):e2510298. doi: 10.1001/jamanetworkopen.2025.10298 (PMC12079294; doi:10.1001/jamanetworkopen.2025.10298)
Supplement: Supplement 2. — Data Sharing Statement [file jamanetwopen-e2510298-s002.pdf]

Data Sharing Statement

Chang. Referral Criteria for Specialist Palliative Care for Patients With Dementia. *JAMA Netw Open*. Published May 14, 2025. doi:10.1001/jamanetworkopen.2025.10298

Data
Data available: No

Additional Information
Explanation for why data not available: The Delphi round outcomes are already presented in the respective tables/supplemental.
